# Short-term outcome of patients with possible transient ischemic attacks: a prospective study

**DOI:** 10.1186/s12883-015-0333-1

**Published:** 2015-05-13

**Authors:** Mariana Correia, Ana Catarina Fonseca, Patrícia Canhão

**Affiliations:** Department of Neurosciences (Neurology), Hospital de Santa Maria, Lisbon, Portugal; Instituto de Medicina Molecular, Lisbon, Portugal; Medical University of Lisbon, Lisbon, Portugal; Serviço de Neurologia, Hospital de Santa Maria, Avenida Professor Egas Moniz, 1649-035 Lisbon, Portugal

**Keywords:** Transient ischemic attacks, Possible TIA, Prognosis, Stroke, Vascular events, Prognosis

## Abstract

**Background:**

Patients with transient ischemic attack (TIA) have an increased risk of vascular events. There is scarce data regarding the prognosis of patients with transient neurological symptoms less typical of TIA, in whom a vascular origin cannot be excluded, also known as possible TIA. In this study we aimed to compare the short-term prognosis between TIA and Possible TIA patients.

**Methods:**

Patients with transient neurological events consecutively referred to a TIA Clinic during five years were classified as TIA, Possible TIA or mimic. Patients were prospectively followed. We compared the outcome at 30 and 90 days after TIA or Possible TIA. The primary outcome was stroke and the secondary outcome was a combination of vascular events (stroke, TIA, myocardial infarction or vascular death).

**Results:**

Two hundred and fifty eight TIA and 109 Possible TIA patients were included. Possible TIA patients had no stroke 30 and 90 days after the event. In contrast, 3.1 % and 4 % of TIA patients had stroke at the same time points. Combined vascular events occurred in 1.9 % of Possible TIA (myocardial infarction) and 9.8 % of TIA patients (stroke and TIA) after 30 days (OR = 0.18, 95 % CI 0.04 to 0.76, *P* = 0.02); and in 1.9 % of Possible TIA patients (myocardial infarction) and 11.3 % of TIA patients (stroke and TIA) after 90 days (OR = 0.16, 95 % CI 0.04 to 0.67, *P* = 0.012).

**Conclusions:**

In this exploratory study, Possible TIA patients had less short-term vascular events than TIA patients.

## Background

The differential diagnosis between transient ischemic attack (TIA) and other transient neurological conditions is challenging. Common TIA mimics such as migraine, transient global amnesia, vertigo, epilepsy, syncope or psychiatric disorder are easy to diagnose [1, 2]. However, many patients with atypical symptoms for TIA or with clinical presentations that do not fulfill the criteria of a specific mimic, remain without a definitive diagnosis [3]. In some of these patients a vascular cause for the symptoms cannot be excluded and these patients are often referred as having a Possible TIA. In a previous study done at our TIA Clinic, about one quarter of patients with transient neurological symptoms were classified as having Possible TIA [4].

TIA patients are at increased risk of early stroke, with a pooled risk of recurrent stroke of 5.2 % at 7 days and 6.7 % at 90 days [5]. TIA patients are also at risk of recurrent TIA (13.4 %) [6] and coronary events (2.9 %) during the following 90 days [7]. However, there is scarce data about the prognosis of patients with Possible TIA. Therefore in this exploratory study, we aimed to evaluate the short-term (30 and 90 days) prognosis of Possible TIA patients and compare it with the prognosis of TIA patients.

## Methods

We included consecutive patients evaluated in the TIA Clinic of Hospital Santa Maria in Lisbon between March 2004 and September 2009. This is a university hospital with Stroke Unit and with access to standard investigations. During recruitment, the TIA Clinic operated once a week. Patients were referred from the Emergency Department (ED) of the hospital. CT scan, ECG and blood analysis were performed in the ED. The remaining etiological studies were requested at the TIA Clinic.

### Procedures

All patients were assessed in the TIA Clinic by a stroke dedicated neurologist (PC). Systematically collected data included age, gender, vascular risk factors, past medical history, description of symptoms (time of onset, type, and duration), presence of simultaneous symptoms (e.g., headache, pain, syncope, anxiety), neurological examination, results of complementary tests (Brain Computerized Tomography (CT) scan or Magnetic Resonance Imaging (MRI), ECG, blood analysis, neck ultrasonography, transcranial Doppler, echocardiography, 24 h Holter monitoring and EEG) and treatment. The ABCD^2^ risk score was calculated for each patient [8]. Recurrent vascular events (TIA, stroke, myocardial infarction (MI) and vascular death) between the transient neurological event and TIA Clinic assessment (first appointment) were registered.

Patients were prospectively reevaluated 30 days (second appointment) and 90 days (third appointment) after inclusion event. If patients missed the clinical appointment they were contacted by telephone. The date and type of vascular recurrence were recorded. Data from the outpatient TIA Clinical, hospital and ED clinical files was also used. If patients did not answer the telephone calls these data sources were used to search for a fatal or non-fatal vascular event. Following the third appointment, patients were discharged to their general physician.

### Classification of transient neurological events

TIA was diagnosed if history and examination were typical for a sudden, focal neurological deficit of presumed vascular origin lasting less than 24 h and there was supportive or no contradictory brain imaging [9]. Possible TIA was considered when clinical symptoms did not fulfill international accepted criteria for TIA (e.g. tiredness or heavy sensation in one or more limbs; isolated disorder of swallowing or articulation, double vision, dizziness, or uncoordinated movements; accompanying symptoms including unconsciousness, limb jerking, tingling of the limbs or lips) [3], and did not fulfill the criteria for a specific mimic diagnosis, although a vascular origin could not be excluded. Mimic was diagnosed according to predefined classification criteria (e.g. aura with or without migraine, seizure, metabolic syndrome, transient global amnesia, conversion, panic attack, syncope).

Two investigators (PC, AF) independently classified the transient neurological events. In the event of disagreement, consensus was reached through discussion. In patients with several repeated episodes, the inclusion episode was the one that motivated the first evaluation by a physician and referral to the TIA Clinic.

Follow-up was considered to start on the day of the inclusion episode.

For the present study, we included patients with TIA and Possible TIA. Patients a priori known to be unable to be followed after the first visit were not included.

### Outcomes

Primary outcome was stroke.

Secondary outcome was a combination of vascular events (myocardial infarction, stroke, TIA or vascular death).

We analyzed outcomes 30 and 90 days after the inclusion event.

### Definition of outcome events

Stroke was diagnosed when a patient presented focal symptoms or signs lasting longer than 24 h and which were confirmed by brain CT scan or MRI.

Myocardial infarction was defined by the presence of the following: typical pain, new electrocardiographic changes [10] and increased troponin levels.

Vascular death was diagnosed in the event of sudden death or when the patient died within one month after stroke or myocardial infarction.

### Statistical analysis

We used descriptive statistics to characterize TIA and Possible TIA patients. Continuous variables were expressed as mean (standard deviation, SD) or median (interquartile range, IQR) and categorical variables as frequencies and percentages.

We compared main clinical characteristics between TIA and Possible TIA patients using χ^2^ tests (with Yates correction when necessary) for categorical variables, and *Mann–Whitney U* test for continuous variables as appropriate.

We compared the frequency of outcomes at 30 and 90 days between TIA and Possible TIA patients using χ^2^ tests (with Yates correction when necessary); we calculated Odds ratios and 95 % confidence intervals (CI).

A level of *P* < 0.05 was considered significant.

Analyses were performed with SPSS for Windows, release 20.0.

This study was in compliance with the Helsinki Declaration and was approved by the local Ethics Committee “*Comissão de Ética para a Saúde”* of the Hospital de Santa Maria. The research involved no procedures that would be different outside the clinical setting. It had no additional risks or costs to the patients. Confidentiality of records was fully guaranteed. Participation in the study was voluntary. This was briefly explained to the all patients in order to obtain an informed verbal consent from potential subjects.

## Results

### Baseline characteristics

Between March 2004 and September 2009, 458 patients with symptoms lasting less than 24 h were referred to the TIA Clinic. Ninety patients had a TIA mimic (23 presyncope or syncope, 16 seizure, 19 psychiatric, 5 migraine, 5 transient global amnesia, 5 vertigo, 6 worsening of previous neurologic deficit, 1 metabolic disturbance, 1 iatrogenic, 1 peripheral nerve compression, 8 non-classifiable). Three hundred and sixty seven patients were included, 258 had the diagnosis of TIA and 109 of Possible TIA; one patient was excluded due to absence of follow-up data.

Main baseline characteristics of both groups (TIA and Possible TIA) are listed in Table 1. Possible TIA patients were younger than TIA patients, had less hypertension and atrial fibrillation and scored lower on the ABCD^2^ score. Regarding clinical symptoms, Possible TIA patients had less aphasia or motor symptoms, more often sensory symptoms and positive features (paresthesias), and frequently described progressive symptoms onset.

**Table 1:** Comparison of main characteristics between transient ischemic attacks (TIA) and possible TIA patients

	TIA	Possible TIA	
	(n = 258)	(n = 109)	*P*
Male gender	154 (59.7 %)	65 (59.6 %)	0.922
Median age (IQR)	68 (59–77)	62 (49–69)	<0.001
Median time (days) between event and visit (IQR)	4.5 (2–7)	4 (2–7)	0.976
Hypertension	189 (73.5 %)	67 (61.5 %)	0.021
Diabetes	44 (17.1 %)	17 (15.6 %)	0.720
Dyslipidemia	121 (47.3 %)	49 (45 %)	0.685
Ischemic heart disease	25 (9.7 %)	10 (9.2 %)	0.878
Atrial fibrillation	23 (8.9 %)	1 (0.9 %)	0.005
Previous TIA	22 (8.5 %)	5 (4.6 %)	0.186
Previous stroke	6 (2.3 %)	6 (5.5 %)	0.194
Smoking			0.536
Ever	174 (67.7 %)	80 (73.4 %)	
Former	44 (17.1 %)	12 (11.0 %)	
Current	39 (15.2 %)	17 (15.6 %)	
Acute onset	241 (93.4 %)	62 (56.9 %)	<0.001
Motor symptoms	146 (56.6 %)	31 (28.4 %)	<0.001
Aphasia	71 (27.7 %)	9 (8.3 %)	<0.001
Dysarthria	41 (16.0 %)	15 (13.8 %)	0.584
Sensory symptoms	86 (33.3 %)	51 (46.8 %)	0.015
Positive symptoms	24 (9.3 %)	24 (22.2 %)	0.001
Duration (minutes) - Median (IQR)	30 (10–120)	30 (8–90)	0.087
ABCD2 score >4	185 (72 %)	36 (34.6 %)	<0.001
Presumed vascular territory			<0.001
Carotid	198 (76.7 %)	38 (35.2 %)	
Vertebro-basilar	52 (20.2 %)	56 (51.9 %)	
Undetermined	8 (3.1 %)	14 (13.0 %)	
TOAST Classification			0.009
Large-vessel atherosclerosis	39 (15.1 %)	6 (5.5 %)	
Cardioembolism	33 (12.8 %)	6 (5.5 %)	
Small vessel occlusion (lacune)	46 (17.8 %)	18 (16.5 %)	
Other determined etiology	8 (3.1 %)	2 (1.8 %)	
Undetermined etiology (≥2 causes, negative or incomplete evaluation)	132 (52.3 %)	82 (75.3 %)	

### Etiological investigation: TIA versus possible TIA

Overall, most ancillary investigation (CT, blood tests, electrocardiogram, transthoracic echocardiogram, neck ultrasonography, transcranial Doppler and transesophageal echocardiogram) was similarly performed on Possible TIA and TIA patients. 24 h ECG monitoring was performed on 112 TIA patients and 27 Possible TIA patients (43.4 % vs. 24.8 %, *P* = 0.001). MRI (17.6 % vs. 8.5 %, *P* = 0.010) and electroencephalogram (11.3 % vs. 4.4 %, *P* = 0.005) were requested more often in Possible TIA patients. A cardioembolic source was identified more often in TIA patients [38 (14.7 %) vs. 6 (5.5 %), *P* = 0.013] as well as internal carotid artery stenosis > 50 [42 (16.6 %) vs. 3 (2.8 %), *P* < 0.001]. The frequency of negative etiological investigation was similar in TIA and Possible TIA patients [110 (42.6 %) vs. 51 (46.8 %), *P* = 0.464].

### Secondary prevention: TIA versus possible TIA

Antiplatelet agents were equally prescribed to TIA and Possible TIA patients [236 (91.8 %) vs. 98 (91.6 %), *P* = 0.94]. Anticoagulation was more frequently prescribed to TIA patients [20 (7.8 %) vs. 2 (1.9 %), *P* = 0.052] but this difference was not significant after adjusting for the presence of cardioembolic disease. Blood pressure lowering agents were more often prescribed to TIA patients [188 (73.4 %) vs. 61 (56.5 %), *P* < 0.001]. Lipid lowering drugs were more often given to TIA patients [213 (83.5 %) vs. 70 (64.8 %), *P* < 0.001].

### Follow-up

At day 2 all patients had follow-up, at day 7–365 patients (99.5 %), at day 30–360 patients (98.1 %) and at day 90–349 patients (95.1 %).

### Primary outcome-stroke

There was a trend for an increase stroke risk in TIA patients compared with Possible TIA patients:

- At Day 30, patients with Possible TIA had no stroke in contrast to 8 (3.1 %) TIA patients who had a stroke (OR = 0.14, 95 % CI 0.01 to 2.38, *P* = 0.172);
- At Day 90, cumulatively no patients with Possible TIA had a stroke and 10 (4.0 %) TIA patients had a stroke (OR = 0.11, 95 % CI 0.01 to 1.90, *P* = 0.129).

### Secondary outcomes-combined vascular events

TIA patients experienced vascular events more often than Possible TIA patients at Day 30 and Day 90:

- At Day 30, two (1.9 %) Possible TIA patients had a vascular event (myocardial infarction, that was fatal in one patient), and 25 (9.8 %) TIA patients suffered a vascular event (eight strokes and 17 TIA) (OR = 0.18, 95 % CI 0.04 to 0.76, *P* = 0.02);
- At Day 90, cumulatively two (1.9 %) Possible TIA patients and 28 (11.3 %) TIA patients suffered a vascular event (10 strokes and 18 TIA) (OR = 0.16, 95 % CI 0.04 to 0.67, *P* = 0.012).

The two Possible TIA patients who suffered vascular events reported unspecific gait disturbance and left facial hyposthesia/dysesthesia respectively in the inclusion episode. Regarding the cardiac investigation of these patients, one had a left atrial enlargement documented in transthoracic echocardiogram, the other had paroxysmal atrial fibrillation (Table 2).

**Table 2:** Cardiac findings in possible TIA patients (n = 109)

Transthoracic Echocardiogram	Myocardial abnormalities	Left atrial enlargement (13) ^+^
		Segmental ventricular disease (5)
		Global myocardial dysfunction (6) ^+^
(n = 79 patients)	Valvular abnormalities	Aortic valve – severe stenosis (1)
ECG/Holter	Arrythmias	Atrial Fibrillation (2) *
(n = 109/27 patients)	Conduction defect	Right bundle branch block (1)
		Left anterior fascicular block (1)
		2nd AV block (2)

Patients with vascular events during the follow-up with * myocardial infarction and sudden vascular death (1 patient) and ^+^ myocardial infarction (1 patient)

## Discussion

In this exploratory study, Possible TIA patients had a low short-term risk of stroke or other vascular events (TIA, MI and vascular death). TIA patients had a frequency of stroke at 30 or 90 days within the range reported by other cohort studies [5].

To our best knowledge the short-term prognosis of patients with Possible TIAs has not been previously reported. The decision to evaluate only the short-term prognosis was based on reports showing that the risk of stroke is particularly high shortly after the TIA [11].

Few studies addressed the prognosis of patients with transient neurological symptoms that do not fulfill criteria for definitive TIA. In the Dutch TIA trial [12], Koudstaal et al. assessed the long-term outcome of patients with atypical symptoms of TIA, who in our classification correspond to Possible TIA. They found higher risk of major cardiac events in patients with atypical TIAs than in patients with typical TIAs and a similar risk of major vascular events in both groups. We would point out that that the two events suffered by our Possible TIA patients were acute myocardial infarctions.

Bos et al. [13] classified transient neurologic attacks as focal, non-focal or mixed (focal and non-focal). In their classification, focal transient neurological attacks were equivalent to TIA. Bos et al. showed that patients with mixed transient neurologic attacks had higher risk of stroke, dementia, ischemic heart disease, and vascular death than patients without transient neurologic attacks. The definition of mixed transient neurologic attack differs from the Possible TIA definition as it was considered by the authors that patients presenting cardiac or vegetative symptoms were classified as mixed. This might create a bias as this group may include a subset of patients in whom cardiac disease is prominent, therefore explaining the high number of vascular events found in the long-term follow-up of these patients. The low risk of stroke of our Possible TIA patients might also be explained by the high rate of treatment with antiplatelets and statins. The risk of stroke within 90 days after focal transient neurological attacks was 3.5 %, similar to our findings in TIA patients.

Our study suggests that the short-term prognosis of Possible TIAs appears to be better than that of TIAs. The low frequency of vascular events that we found at short-term after Possible TIA can have several possible explanations. First, Possible TIA patients seem to be a different group compared with TIA patients. They have less vascular risk factors, lower ABCD^2^ score, less cardioembolic disease or severe atherothrombotic carotid artery disease. In consequence, at short-term they may have lower risk of vascular events. Second, Possible TIA patients might not have a vascular cause for the symptoms and therefore they might have lower risk for stroke. It should be noted that event classification was done prior to knowing the vascular risk profile and the results of the etiological study, therefore there was no possible classification bias [4]. Third, because this study was carried out when the TIA Clinic functioned once a week, we cannot exclude a referral bias caused by referral of patients with low risk, nor that some Possible TIA patients might have had an outcome event before the TIA Clinic appointment.

One of the major limitations of this study was infrequent magnetic resonance imaging. About 10 % of patients in the acute phase did MRI. We cannot be sure if the presence of DWI ischemic lesions would have changed the diagnosis of Possible TIA into TIA. However, a recent systematic review showed that about two thirds of patients with definite specialist-confirmed TIA have negative DWI findings [14]. It could be important to know the results of the MRI in Possible TIAs, but ultimately the diagnosis of transient neurological events remains clinical, and the absence of acute ischemic lesion in DWI would not have changed the diagnosis of TIA or Possible TIA. The degree of certainty whether a patient has a TIA or Possible TIA is based on clinical judgment, the experience of the physician and the patient’s ability to explain the event.

## Conclusions

About one fourth of patients with transient neurological symptoms referred to a TIA Clinic were diagnosed as having a Possible TIA. Possible TIA patients had lower short-term risk of vascular events (stroke, TIA, myocardial infarction or vascular death) than TIA patients. Overall, Possible TIA patients were managed and treated like TIA patients because a vascular origin of the symptoms could not be excluded. It is possible that the high intensity treatment with antiplatelets and statins might be responsible for an even lower event rate in Possible TIA patients.

Our findings are important because little data exists concerning this particular group of patients and because they are frequently encountered in common clinical practice. It would be important to replicate our results with larger samples and in different clinical settings.
